# Comparative genomic analysis of *Parageobacillus thermoglucosidasius* strains with distinct hydrogenogenic capacities

**DOI:** 10.1186/s12864-018-5302-9

**Published:** 2018-12-06

**Authors:** Teresa Mohr, Habibu Aliyu, Raphael Küchlin, Michaela Zwick, Don Cowan, Anke Neumann, Pieter de Maayer

**Affiliations:** 1 Section II: Technical Biology, Institute of Process engineering in Life Science, Karlsruhe Institute of Technology, Kaiserstrasse 12, 76131 Karlsruhe, Germany; 2 Centre for Microbial Ecology and Genomics, Department of Biochemistry, Genetics and Microbiology, University of Pretoria, Hatfield, Pretoria, 0028 South Africa; 3 School of Molecular & Cell Biology, Faculty of Science, University of the Witwatersrand, WITS, Johannesburg, 2050 South Africa

**Keywords:** Biohydrogen production, *Parageobacillus thermoglucosidasius*, Water-gas shift reaction, Comparative genomics, DSM 6285

## Abstract

**Background:**

The facultatively anaerobic thermophile *Parageobacillus thermoglucosidasius* produces hydrogen gas (H_2_) by coupling CO oxidation to proton reduction in the water-gas shift (WGS) reaction via a carbon monoxide dehydrogenase–hydrogenase enzyme complex. Although little is known about the hydrogenogenic capacities of different strains of this species, these organisms offer a potentially viable process for the synthesis of this alternative energy source.

**Results:**

The WGS-catalyzed H_2_ production capacities of four distinct *P. thermoglucosidasius* strains were determined by cultivation and gas analysis. Three strains (DSM 2542^T^, DSM 2543 and DSM 6285) were hydrogenogenic, while the fourth strain (DSM 21625) was not. Furthermore, in one strain (DSM 6285) H_2_ production commenced earlier in the cultivation than the other hydrogenogenic strains. Comparative genomic analysis of the four strains identified extensive differences in the protein complement encoded on the genomes, some of which are postulated to contribute to the different hydrogenogenic capacities of the strains. Furthermore, polymorphisms and deletions in the CODH-NiFe hydrogenase loci may also contribute towards this variable phenotype.

**Conclusions:**

Disparities in the hydrogenogenic capacities of different *P. thermoglucosidasius* strains were identified, which may be correlated to variability in their global proteomes and genetic differences in their CODH-NiFe hydrogenase loci. The data from this study may contribute towards an improved understanding of WGS-catalysed hydrogenogenesis by *P. thermoglucosidasius*.

**Electronic supplementary material:**

The online version of this article (10.1186/s12864-018-5302-9) contains supplementary material, which is available to authorized users.

## Background

Members of the genus *Parageobacillus* are Gram-positive, facultatively anaerobic thermophiles belonging to the family *Bacillaceae* and the phylum Firmicutes [1]. They are readily isolated from a wide range of high temperature environments including hot springs, deep oil wells and desert soils [2]. The thermophilic nature of this genus has resulted in considerable interest in *Parageobacillus* as a source of a broad range of industrially relevant, thermostable enzymes, such as lipases, proteases and hemicellulases [3–5]. Furthermore, there has been increasing interest in the use of *Parageobacillus* spp. as whole cell biocatalysts in a broad of biotechnological applications, such as the production of bioethanol, the biorefinement of linen fibres and the bioremediation of environmental pollutants [6–8].

The biotechnological value of *Parageobacillus* spp. can partly be attributed to the expansive metabolic capacities of members of this genus, which effectively utilize a broad range of complex polysaccharides and oligosaccharides for growth, including hemicellulose [3], hydrocarbons and aromatic compounds [3, 9, 10]. Furthermore, *Parageobacillus* spp. can grow anaerobically, where they produce lactate, formate, acetate, ethanol and succinate using mixed acid fermentation pathways [7]. Recently, we showed that the type strain of *Parageobacillus thermoglucosidasius* is also able to produce hydrogen gas (H_2_) in the anaerobic phase following aerobic growth, concomitant with the consumption of carbon monoxide (CO) [11]. Genomic analysis linked this capacity to a genetic locus comprising of three genes coding for a carbon monoxide dehydrogenase (CODH) and 12 genes coding for a NiFe group 4a hydrogenase. This CODH-NiFe hydrogenase complex catalyses the water-gas shift (WGS) reaction, where CO is oxidized by CODH with the resultant electrons being used for the reduction of protons by the NiFe hydrogenase, resulting in production of H_2_ (CO + H_2_O → CO_2_ + H_2_) [11, 12].

In recent decades, the need for reducing the use of conventional energy sources and the use of so called ‘green power’ has received increasing attention [13, 14]. Hydrogen gas has been extensively studied as alternative energy source as it carries the highest energy per unit mass, can be stored easily and its combustion results in the release of water vapour, making it cost effective and environmentally ‘friendly’ [15, 16]. However, a significant hurdle for the use of H_2_ as alternative energy carrier is the currently available production practice [17]. H_2_ is largely produced by industrial means, including through steam reformation of methane, coal gasification and electrolysis of water, all of which are costly and are often detrimental to the environment [17]. There has thus been increasing interest in the development of biological H_2_ production processes [16]. Bacteria using the WGS reaction show considerable promise in this regard, given that CO is a component of “syngas” (comprising primarily of CO, CO_2_ and H_2_) resulting from a wide range of industrial processes, including the gasification of coal [18]. However, the majority of bacteria using the WGS are strictly anaerobic, implying that oxygen (O_2_) would first have to be removed from the gas mixture, at high cost [18]. *P. thermoglucosidasius* would be a promising candidate for further exploration as it can grow aerobically and, once O_2_ has been consumed, can shift to the anaerobic WGS reaction [11, 12]. In the current study, the ability of four distinct *P. thermoglucosidasius* strains to produce H_2_ via the WGS reaction was analysed. Three of the four strains were hydrogenogenic and the hydrogenogenic strains showed differences in the time taken to start H_2_ production. Comparative genomic approaches were applied to identify the potential molecular basis for the variable hydrogenogenic capacities.

## Results

### *P. thermoglucosidasius* strains vary in their ability to produce hydrogen

**Fig. 1:**
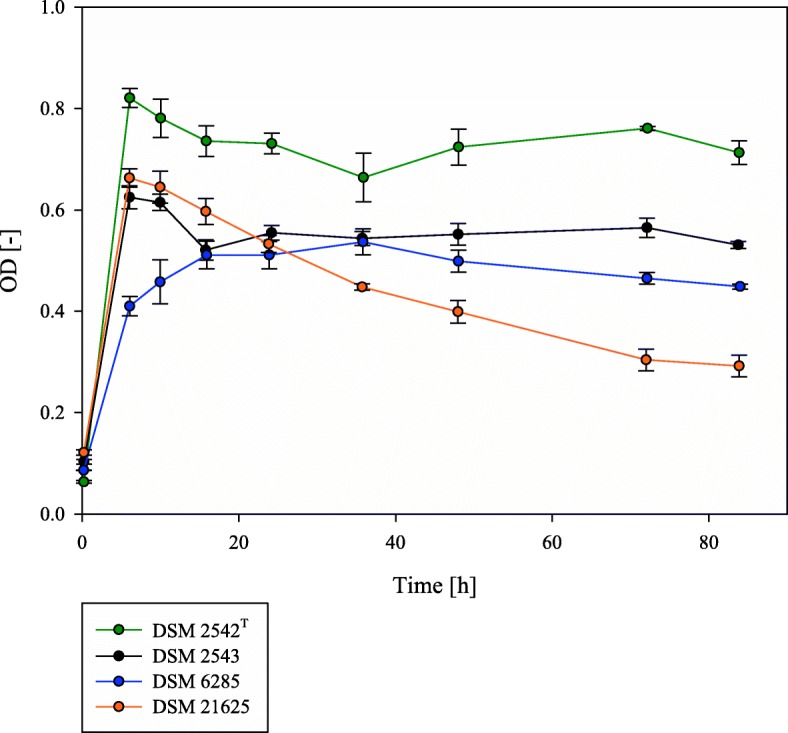
Growth curves of four *P. thermoglucosidasius* strains. The strains were cultivated in mLB medium in stoppered serum bottles with an initial gas atmosphere consisting of 50% CO and 50% air. DSM 2542^T^ (green) and DSM 2543 (black) reached their maximum absorbance after ~ 6 h while there was still O_2_ present. DSM 6285 (blue) reached its maximum absorbance OD_600_ = 0.537 ± 0.02 after ~ 36 h during the anaerobic phase. The non hydrogenogenic strain DSM 21625 (orange) reached its maximum (OD_600_ = 0.645 ± 0.032) after 9.39 h and decreased to a final value of OD_600_ = 0.292 ± 0.021 at the end of the cultivation

Four *P. thermoglucosidasius* strains, DSM 2542^T^, DSM 2543, DSM 6285 and DSM 21625, were cultivated (in quadruplicate) for a total duration of 84 h in stoppered 250 ml flasks containing 50 ml of modified Luria Bertani (mLB) medium and an initial gas atmosphere of 50% CO and 50% air. The volume percentage of gases, CO, CO_2_, O_2_ and H_2_, were routinely monitored using gas chromatography (GC) analysis. All four strains were able to grow in the presence of CO, but reached a maximum absorbance at different time points in the cultivation (Fig. 1). Two strains, DSM 2542^T^ (OD_600_ = 0.821 ± 0.019) and DSM 2543 (OD_600_ = 0.625 ± 0.023), reached maximum absorbance after ~ 6 h, while DSM 21625 reached a maximum absorbance (OD_600_ = 0.645 ± 0.032) ~ 10 h after inoculation. By contrast *P. thermoglucosidasius* DSM 6285 reached a maximum absorbance only after ~ 36 h (OD_600_ = 0.537 ± 0.026). In all four strains, O_2_ was consumed ~ 24 h post inoculation, plateauing at a final value of 0.278 ± 0.007 mmol (Additional file 1). While three strains reached their maximum absorbance while O_2_ was still present, the slower growing *P. thermoglucosidasius* DSM 6285 reached its maximum absorbance nearly 12 hours after O_2_ was depleted (Additional file 1). This suggests that this strain possesses the metabolic capacity to support fully anaerobic growth. For two of the faster-growing strains, DSM 2542^T^ and DSM 2543, a gradual recovery in absorbance was observed following the decline after maximal growth. By contrast, for the fourth strain (*P. thermoglucosidasius* DSM 21625) the absorbance continued to decline following O_2_ consumption (Fig. 1).

**Fig. 2:**
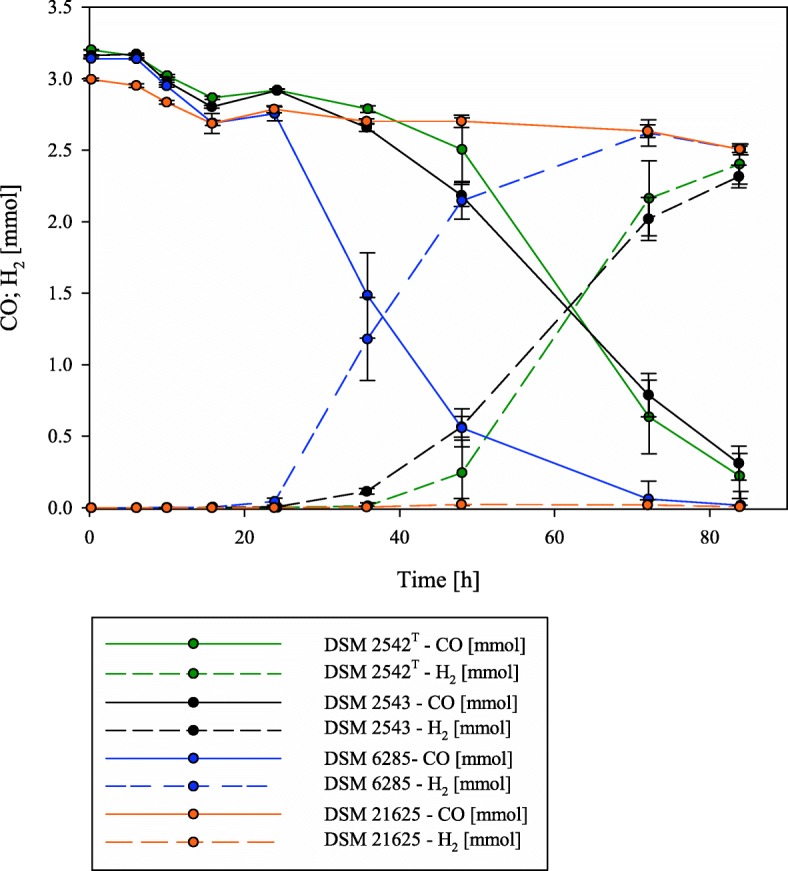
CO consumption and H_2_ production of the hydrogenogenic strains during the cultivation with an initial gas atmosphere of 50% CO and 50% air. DSM 2542^T^ (green) and DSM 2543 (black) started to produce H_2_ after ~ 36 h (dotted lines). They achieved a final yield of 1.08 CO/H_2_ (DSM 2542^T^) and 0.95 CO/H_2_ (DSM 2543). *P. thermoglucosidasius* DSM 6285 (blue) started the hydrogen production already after ~ 16 h. For DSM 21625 no hydrogen was detected (orange)

Analysis of the gas compositions during the cultivation revealed key differences between the four strains. For three of the strains, DSM 2542^T^, DSM 2543 and DSM 6285, H_2_ was produced with the concomitant consumption of CO after O_2_ reached its minimal plateau level (Fig. 2; Additional file 1). By contrast, while a nominal decrease in the amount of CO (0.302 ± 0.373 mmol) could be observed, no H_2_ was produced by *P. thermoglucosidasius* DSM 21625 throughout the cultivation (Additional file 1). In the three hydrogenogenic strains, the commencement of H_2_ production coincided with a slight increase in absorbance observed in the growth curve (Fig. 1). This suggests that the WGS reaction plays a role in the continued growth of these strains under anaerobic conditions. This is supported by the continued decline in absorbance observed for DSM 21625, which was unable to produce H_2_ when exposed to CO.

Only minor differences were observed in terms of the H_2_ produced and CO consumed after 84 h, for DSM 2542^T^ (H_2_ produced: 2.470 ± 0.149 mmol; CO consumed: 2.280 ± 0.11 mmol), DSM 2543 (H_2_ produced: 2.389 ± 0083 mmol; CO consumed: 2.512 ± 0.106) and DSM 6285 (H_2_ produced: 2.637 ± 0.058 mmol; CO consumed: 2.552 ± 0.058 mmol), with an average yield of 1.02 H_2_/CO (Additional File 1). There was, however, an observable difference in the time taken by the hydrogenogenic strains to start utilizing CO and produce H_2_. Whereas DSM 2542^T^ and DSM 2543 initiated H_2_ production after ~ 36 h, H_2_ production by DSM 6285 commenced ~ 16 h after inoculation (i.e., the lag phase between growth phase and H_2_ production was substantially shorter for *P. thermoglucosidasius* DSM 6285). In order to further characterise the different hydrogenogenic capacities of the *P. thermoglucosidasius* strains, and the faster onset of H_2_ production by *P. thermoglucosidasius* DSM 6285 compared to the other two hydrogenogenic strains, the genomes of the four strains were sequenced and compared using in silico methodologies.

### Comparative genomics reveals substantial genome diversification among the compared *P. thermoglucosidasius* strains

**Fig. 3:**
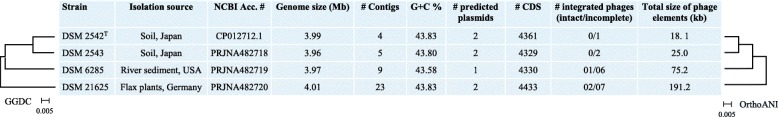
Genome properties of the compared *P. thermoglucosidasius* strains. The isolation source, genome size, number of contigs, G + C % and number of proteins encoded on the genome are indicated. Similarly, the number of predicted plasmids and integrated phages are shown. The dendrograms at either end show the phylogenetic relationships of the strains on the basis of digital DNA-DNA hybridization values (left) and OrthoANI values (right), respectively

The genomes of *P. thermoglucosidasius* DSM 2543, DSM 6285 and DSM 21625 were assembled to high quality draft status of between five and 22 contigs. The complete genome sequence of *P. thermoglucosidasius* DSM 2542^T^ is comprised of four replicons. The genomes of the four strains range in size between 3.96 and 4.01 Mb with an average G + C content of 43.76% (Fig. 3).

DSM 6285 harbours one plasmid while the other three strains have two plasmids. Between 4329 (DSM 2543) and 4433 (DSM 21625) proteins are encoded on the genomes. The genomic relatedness of the four strains was determined by calculating the digital DNA-DNA hybridization (GGDC) [19] and OrthoANI [20] values for each paired combination of strains. This showed that *P. thermoglucosidasius* DSM 2542^T^ and DSM 2543, isolated from the same environmental source, were most closely related [21], while DSM 21625 was the most distinct strain on the basis of these two genomic values (Fig. 3). However, both GGDC (> 70%) and ANI (> 95%) values exceed those distinguishing distinct species, confirming that all four strains belonged to the species *P. thermoglucosidasius* (Additional file 2).

**Fig. 4:**
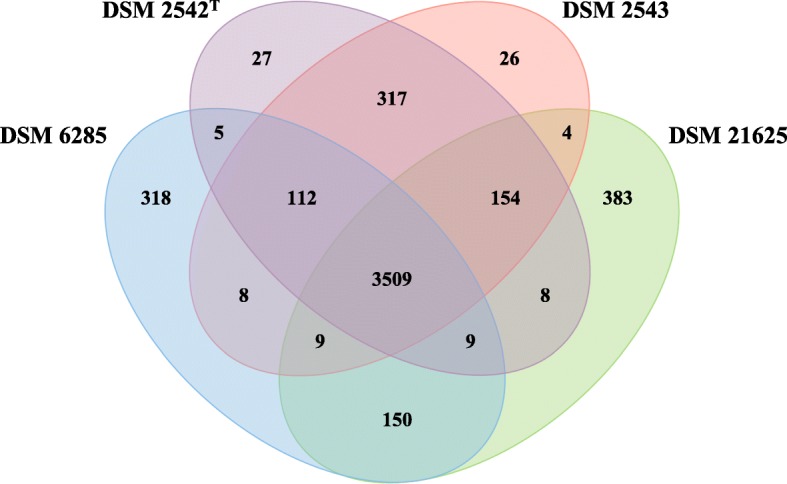
Venn diagram of protein families shared among or unique to the four compared *P. thermoglucosidasius* strains

The proteins encoded on the genomes of the four *P. thermoglucosidasius* strains were compared pair-wise using Orthofinder [22]. This analysis showed that the total protein content of the genomes comprised 5039 distinct protein families (Fig. 4). Of these 3509 (69.63%) constituted the core protein families shared among all four strains. This core protein family dataset contributes between 83.03 (DSM 21625) and 85.17% (DSM 6285) of the total protein families present on each genome. When considering the unique protein families for each of the strains, the two most closely related strains, *P. thermoglucosidasius* DSM 2542^T^ and DSM 2543, contained the smallest fraction of strain-unique proteins (< 0.65% of total protein families) (Fig. 4). *P. thermoglucosidasius* DSM 2542^T^ and DSM 2543 did, however, have a large shared fraction (317 protein families) which was not found in the other two strains. Larger strain-unique protein fractions were observed for DSM 6285 (7.72%) and DSM 21625 (9.06%) (Fig. 4). These differences can be largely attributed to the integration of several prophages within the genomes of these two strains, with phage elements contributing ~ 1.89 and 4.77% of the total genomic DNA of *P. thermoglucosidasius* DSM 6285 and DSM 21625, respectively.

### Differences in the proteome may contribute to the variable H_2_ production capacities of the *P. thermoglucosidasius* strains

The core and accessory protein datasets of the four *P. thermoglucosidasius* strains were compared to assess whether the distinctive H_2_ production capacities might be correlated to differences in their protein complement.

A total of 383 protein families are unique to the non-hydrogenogenic strain (DSM 21625), while 112 protein families are restricted to the hydrogenogenic strains (DSM 2542^T^, DSM 2543 and DSM 6285) (Fig. 4; Additional file 3). Functional annotation and classification according to Conserved Orthologous Groups (COGs) [22] showed that in both cases the datasets are largely comprised of proteins belonging to the COG functional category S (function unknown), with 73.63% (282 proteins) and 76.79% (86 proteins) of the proteins in the non-hydrogenic and hydrogenic dataset, respectively, belonging to this category (Additional file 3). Most of the remaining proteins unique to the non-hydrogenogenic *P. thermoglucosidasius* DSM 21625 are involved in carbohydrate transport and metabolism (G – 9.14%), DNA replication, recombination and repair (L – 5.22%) and transcription (K – 3.39%) (Additional file 3). The majority of proteins in COG category G are encoded by the hemicellulose utilization system (HUS) locus, which has previously been identified as a highly variable locus among members of the genera *Geobacillus* and *Parageobacillus*, encoding a broad range of enzymes and metabolic pathways for the degradation of distinct hemicellulose polymers [3]. Proteins linked to the COG category L include phage primases, endonucleases and terminases, a product of the large number of unique phage elements in this strain. Proteins that form part of an L-arabinose transporter (AraFGH) were unique to the hydrogenogenic strains, located within the HUS locus as well as a branched amino acid transporter (LivFGMHJ) [3].

The shorter H_2_ production lag phase for *P. thermoglucosidasius* DSM 6285 suggests that this strain reaches the metabolic state suitable for the WGS reaction sooner than the other hydrogenogenic strains. Analysis of the unique protein family complement of this strain indicated that the majority of the 468 proteins not shared with DSM 2542^T^ and DSM 2543 belong to the COG category S (function unknown – 76.50%). Considering the proteins in other COG categories, only 24 proteins are involved in metabolic functions, including carbohydrate (G; 5 proteins), amino acid (E; 8 proteins) and inorganic ion transport and metabolism (P; 7 proteins), secondary metabolite biosynthesis, transport and catabolism (Q; 2 proteins) and energy production and conversion (C; 3 proteins) (Additional file 3). Among these metabolic proteins, four are involved in the synthesis of an inorganic ion ABC transporter (NCBI Acc. # DV713_01765–01780). The presence of conserved domains in DV713_01765 (CD08492: PBP2_NikA_DppA_OppA_like_15; E-value: 0e+ 00), DV713_01770 (TIGR02789: NikB; E-value: 4.52e-77), DV713_01775 (TIGR02790: NikC; E-value: 2.38e-67) and DV713_01780 (TIGR02770: NikD; E-value: 2.93e-79) suggest that this may represent a nickel transport system [23]. Nickel is pivotal for the functioning of both anaerobic CODH and Ni-Fe hydrogenases, forming part of the metallocenter of both these enzymes [23].

Also unique to this strain are three proteins involved in the biogenesis of cytochrome *caa3* oxidase. Cytochrome *caa*_*3*_ oxidase is the major oxidase involved in the last stages of the respiratory electron transport chain in *B. subtilis* grown under aerobic conditions, transferring electrons from the cytochrome *c* in the respiratory chain to the terminal electron acceptor, oxygen [24, 25]. Deletion of the structural genes for cytochrome *caa3* oxidase in *B. subtilis* showed that this enzyme is not essential for growth [26]. The unique presence of orthologues of three proteins which are central to cytochrome *c* oxidase biosynthesis in *P. thermoglucosidasius* DSM 6285 may imply that this strain could more efficiently oxidise cytochrome *c* and reduce O_2_ to H_2_O, thereby reaching the critical oxygenic limits for functioning of the anaerobic CODH-hydrogenase enzymes faster than the other strains. However, comparison of the O_2_-consumption rates of the hydrogenogenic strains did not show any substantial difference in terms of the time taken until O_2_ reached its minimum. Differences at the gene level, particularly in the CODH-NiFe hydrogenase loci, may also contribute to the disparity in the hydrogenogenic capacities of the *P. thermoglucosidasius* strains.

### Variation in the CODH-hydrogenase locus of hydrogenogenic and non-hydrogenogenic *P. thermoglucosidasius* strains

**Fig. 5:**
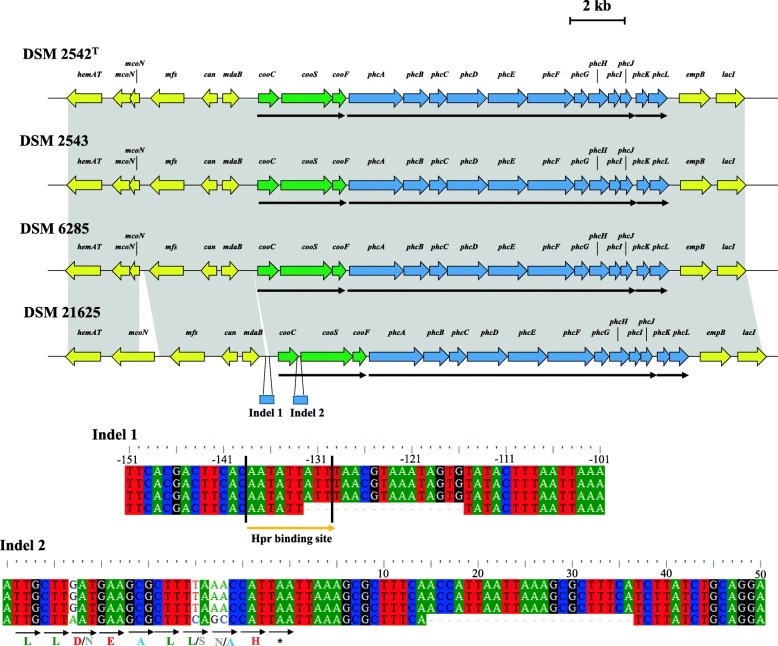
Schematic diagram of the CODH-NiFe group 4a hydrogenase locus of the compared *P. thermoglucosidasius* strains. Genes involved in the synthesis of the CODH are represented by green arrows, while blue arrows indicate those genes required for the synthesis and functioning of the NiFe group 4a hydrogenase. Flanking genes are coloured in yellow. Black arrows below each locus indicate the operonic structure of the CODH-NiFe group 4a hydrogenase loci. The inset shows alignments of the indels occurring upstream and downstream of the *cooC* gene in *P. thermoglucosidasius* DSM 21625. The orange arrow below the Indel 1 alignment indicates the consensus sequence for the Hpr transcription factor binding site. *cooC*, *cooS* and *cooF* code for a CO dehydrogenase maturation factor, *phcA-phcL* code for an H_2_ evolving hydrogenase

In order to further distinguish genomic differences underlying the divergent hydrogenogenic capacities of the four *P. thermoglucosidasius* strains, the CODH-NiFe group 4a hydrogenase loci responsible for CO-oxidation dependent hydrogenogenesis [12] were analysed at both the gene and protein level. In all four strains, the locus encodes three proteins (CooCSF) for the assembly of the CODH enzyme, and 12 proteins (PhcABCDEFGHIJKL) which comprise the NiFe group 4a hydrogenase (Fig. 5). In silico analysis of the operon structure of this locus using FgenesB [27] showed that the genes form part of three distinct operons, *cooCSF, phcABCDEFGHIJ* and *phcKL,* in all four strains (Fig. 5).

To determine whether mutations within the CODH-NiFe hydrogenase genes might be responsible for the difference observed in hydrogenic capacities of the four *P. thermoglucosidasius* strains, the nucleotide sequences for each of the genes in the CODH-NiFe hydrogenase loci of the four strains were aligned and compared. In total, 72 Single Nucleotide Polymorphisms (SNPs) were identified across the 15 genes, with an average of 4.8 SNPs per gene. SNPs were interspersed across the genes rather than clustered together (Additional file 4). More SNPs was observed in the *cooC* (10 SNPs) and *cooS* (11 SNPs) genes, coding for the CODH maturation factor and CODH catalytic subunit, respectively, as well as *phcA* (8 SNPs), *phcB* (13 SNPs) and *phcF* (9 SNPs), which encode the NiFe group 4a hydrogenase component B, membrane subunit and large subunit, respectively. When comparing the different strains, 45 SNPs (62.5% of the total SNPs) were restricted to the non-hydrogenogenic *P. thermoglucosidasius* DSM 21625, with most of these occurring in the *cooC* (10 SNPs), *cooS* (9 SNPs) and *phcF* (8 SNPs) genes, respectively (Additional file 4). A further 14 SNPs were found in the genes of both DSM 21625 and DSM 6285, while 14 SNPs are only found in the hydrogenogenic *P. thermoglucosidasius* DSM 6285. When the proteins encoded by each of the genes were compared, it was observed that the SNPs resulted in only 29 non-synonymous mutations at the amino acid level (Additional file 4), the majority of which occurred in the proteins of DSM 21625 (19–65.72% of the total non-synonymous mutations), with most occurring in CooC (6 mutations) and PhcF (4 mutations). Six distinct non-synonymous mutations were also observed in DSM 6285, which initiates H_2_ production more rapidly than the other two hydrogenogenic strains.

Average amino acid identity values were calculated for the CODH-NiFe hydrogenase protein datasets. The three hydrogenogenic strains share an average amino acid identity of 99.87% across the 15 proteins. The proteins of the non-hydrogenogenic *P. thermoglucosidasius* DSM 21625 shared 99.50% average amino acid identity with those of the hydrogenogenic strains, indicating that this strain was the most divergent. The highest divergence was observed for CooC, where the DSM 21625 protein shared 97.64% average amino acid identity with the orthologous protein in the other three strains, across 254 amino acids.

Alignment of the entire locus using Mauve v2.3.1 [28] revealed the presence of two deletions associated with the intergenic regions of the CODH-hydrogenase locus of DSM 21625, which are not observed in the loci of the three hydrogenogenic strains (Fig. 5). A 22 nucleotide deletion occurs in the intergenic region between *cooC* and *cooS*, 14 nucleotides downstream of the stop codon of *cooC*. The second deletion of 17 nucleotides occurred 115 nucleotides upstream of the start codon of *cooC* (and thus upstream of the CODH-NiFe hydrogenase locus). Putative transcription factor binding sites (TFBSs) were identified in a 500 base pair window upstream of the *cooC* start codon using the TFSITESCAN tool [29]. One predicted TFBS shared homology with the binding site for the *B. subtilis* transition state regulator Hpr [30–32]. Alignment of the flanking regions of the *P. thermoglucosidasius* CODH-NiFe hydrogenase loci showed this transcription factor binds between 139 and 129 bp upstream of *cooC* and the last three nucleotides of this TFBS forms part of the 17 nucleotide deletion in *P. thermoglucosidasius* DSM 21625 (Fig. 5). The deletion within the Hpr binding site might thus explain the lack of H_2_ production in this strain. However, further laboratory analysis is required to identify the regulon for the CODH-NiFe hydrogenase locus to confirm this hypothesis.

## Discussion

The ability of four different *P. thermoglucosidasius* strains to produce hydrogen via the WGS reaction was evaluated. Our analysis revealed extensive differences in the hydrogenogenic capacities of the strains. In particular, *P. thermoglucosidasius* DSM 21625 was unable to produce H_2_ even though a CODH-NiFe hydrogenase locus was shown to be present on the genome. This suggests that the ability to produce H_2_ via the WGS reaction is not a universal trait among *P. thermoglucosidasius* strains. We identified one strain, *P. thermoglucosidasius* DSM 6285, with ‘superior’ hydrogenogenic capacity, with the initiation of H_2_ production after a shorter lag phase than for the other hydrogenogenic strains.

Comparative genomic analyses revealed a number of key differences at the molecular level that may underlie the distinct hydrogenogenic capacities observed for the different *P. thermoglucosidasius* strains. These include an extensive protein set which was unique to the hydrogenogenic strains, and differences in the protein complement of DSM 6285 and the other hydrogenogenic strains. The lack of clear phenotypic differences that can be linked to the variation at the protein level suggests that there may be other factors underlying the differences observed in H_2_ production times for DSM 6285, DSM 2542^T^ and DSM 2543. For example, it is possible that some of the proteins assigned to COG category S (unknown function) play a role in these variable phenotypes. Similarly, proteins of unknown function among the protein families unique to the non-hydrogenogenic *P. thermoglucosidasius* DSM 21625 and unique to the hydrogenogenic strains DSM 2542^T^, DSM 2543 and DSM 6285 may also have an effect on the ability of the different strains to produce hydrogen.

Furthermore, SNPs in the CODH-NiFe hydrogenase loci, and the associated amino acid mutations and deletions in and adjacent to this locus, may also be responsible for the difference in hydrogenogenic phenotype. In particular, a deletion was observed in the binding site for the transition state regulator Hpr upstream of the CODH-NiFe hydrogenase locus on the non-hydrogenogenic strain *P. thermoglucosidasius* DSM 21625. In *B. subtilis*, Hpr has been shown to play a role in the up- and down-regulation of a range of genes involved in post-exponential phase processes such as motility, extracellular enzymes synthesis, antibiotic production and sporulation [30–32]. As the consumption of CO and production of H_2_ by the three H_2_-producing *P. thermoglucosidasius* strains occurs in the post-exponential phase, a role for an Hpr-like regulator in the control of this capacity is plausible.

It cannot be excluded that factors other than observable genetic differences may underlie these distinct phenotypes. For example, the shorter lag phase between aerobic growth and the WGS-driven H_2_ production may be due to differences in the O_2_ sensitivity of the CODH-hydrogenase complex of the hydrogenogenic strains. Proteomic, gene expression and biochemical analyses could shed further light on the phenotypic differences observed in this study.

## Conclusions

*P. thermoglucosidasius* strains differ in their capacity to produce H_2_ via the CODH-NiFe hydrogenase-catalyzed WGS reaction. This may be correlated to extensive differences we observed in terms of the proteins encoded on the genomes of the strains, as well as to SNPs in the CODH-NiFe hydrogenase loci. Further gene expression, proteomic and physiological characterization will be undertaken to elucidate the factors underlying the distinct hydrogenogenic phenotypes. This data will be crucial in the selection of *P. thermoglucosidasius* strains and the optimization of fermentation conditions for incorporation in bioindustrial hydrogen production strategies.

## Methods

### Bacterial strains and culturing conditions

To verify the production of H_2_ of different *P. thermoglucosidasius* strains, four strains *P. thermoglucosidasius* DSM 2542^T^, *P. thermoglucosidasius* DSM 2543, *P. thermoglucosidasius* DSM 6285 and *P. thermoglucosidasius* DSM 21625 were grown in presence of CO. All strains were obtained from the DSMZ (Deutsche Sammlung von Mikroorganismen und Zellkulturen GmbH, Braunschweig, Germany).

The cultivation of the tested strains was conducted as previously described [12]. Briefly, pre-cultures and experimental cultures were grown in mLB medium (modified Luria-Bertani): tryptone (1% *w*/*v*), yeast extract (0.5% w/v), NaCl (0.5% w/v), 1.25 ml/L NaOH (10% w/v), and 1 ml/L of each of the filter-sterilized stock solutions: 1.05 M nitrilotriacetic acid, 0.59 M MgSO_4_.7H_2_O, 0.91 M CaCl_2_.2H_2_O and 0.04 M FeSO_4_.7H_2_O. A first set of pre-cultures was grown aerobically at 60 °C and 120 rpm (24 h). A second pre-culture was inoculated to an OD_600_ = 0.1 from pre-culture 1 and incubated aerobically for 12 h. The cultivations were conducted in serum bottles (250 ml) with 50 ml medium and an initial gas atmosphere consisting of 50% CO and 50% air at 1 bar atmospheric pressure. The bottles were inoculated with 1 ml of the second pre-culture. All cultivations were undertaken at 60 °C and 120 rpm in an Infors Thermotron (Infors AG, Bottmingen, Switzerland) The experiments ran for 84 h and were performed as quadruplicates in stoppered bottles.

### Analytical methods

The gas compositions and culture growth were monitored at nine different time points during the experimental cultivation. For monitoring the growth, 1 ml of the culture was measured at OD_600_ using an Ultrospec 1100 pro spectrophotometer (Amersham Biosciences, USA). The gas composition was monitored at each time point using a 300 Micro GC gas analyzer (Inficon, Bad Ragaz, Switzerland) with the columns Molsieve and PLOT Q. Before and after taking the liquid and gas samples the pressure in the serum bottles was measured using a manometer (GDH 14 AN, Greisinger electronic, Regenstauf, Germany). Gas analysis and calculation of the gas composition were performed as previously described [12].

### Genome sequencing, assembly and annotation

*P. thermoglucosidasius* DSM 2543, DSM 6285 and DSM 21625 were grown aerobically in mLB medium (60 °C; 120 rpm) to mid-log phase. Total DNA was extracted using Quick-DNA™ Fungal/Bacterial Miniprep Kit (Zymo Research, Irvine, CA, USA). The genome of *P. thermoglucosidasius* DSM 2542^T^ was sequenced previously (NCBI Acc. #: CP012712.1). Genome sequencing of the other three strains was conducted using the Illumina Hiseq platform at GATC Biotech (Konstanz, Germany). A total of 9,152,896 (1.38 Gb: ~ 353x coverage), 9,467,702 (1.43 Gb: ~362x coverage) and 9,684,759 (1.46 Gb: ~ 369x coverage) paired reads were generated for *P. thermoglucosidasius* DSM 2543, DSM 6285 and DSM 21625, respectively. De novo genome assembly was undertaken using SPAdes genome assembler v3.11.1 [33] and the resulting contigs were further assembled (scaffolded) with the aid of Medusa v1.6 [34] and CSAR [35] using all available complete genome sequences of *P. thermoglucosidasius* as reference. The plasmids of *P. thermoglucosidasius* DSM 2542^T^ were missing from the available complete genome sequence but were obtained from a second available draft genome of this strain (NCBI Acc. # LAKX01000000).

The high quality draft genome sequences of all four strains were structurally and functionally annotated using the Rapid Annotation RAST using Subsystems Technology (RAST v. 2.0) server [36]. Putative integrated bacteriophages were identified using the Phast server [37]. The genomic relatedness of the four strains was determined using the Genome-to-Genome Distance calculator (GGDC 2.0) [18] and OrthoANI 0.93 [19].

### Comparative genomic analyses

The protein datasets predicted by RAST for all four strains were compared using Orthofinder 1.1.4 [22] with default parameters. This allowed for the identification of protein families (orthologous proteins) found in all four strains (core), shared by two or three strains or unique to individual comparator strains (accessory). Both the core and accessory protein family datasets were functionally annotated by comparison against the EggNOG database (v. 4.5.1) using eggnog-mapper and the NCBI Conserved Domain Database using Batch CD-search [38, 39].

To identify variation in the CODH-NiFe group 4a hydrogenase loci of the four compared strains, these regions were extracted from the genome sequences and compared using Mauve v2.3.1 [28]. SNPs in the genes in this locus were identified by pair-wise alignment of each gene using ClustalW in Bioedit v. 7.2.6 [40, 41]. The operon structures of the CODH-NiFe group 4a hydrogenase loci were determined in silico using FgenesB [27]. Further, transcription factor binding sites (TFBSs) were identified using the TFSITESCAN tool [29].

## Additional files

Additional file 1: Growth curve and gas composition during the cultivation of *P. thermoglucosidasius*), DSM 2542^T^ (A), DSM 2543 (B) and DSM 6285 (C), DSM 21625 (D). All strains were cultivated in quadruplicate in mLB medium with an initial gas atmosphere consisting of 50% CO and 50% air for 84 h. (PDF 261 kb)

Additional file 2: Genomic relatedness among the four compared *P. thermoglucosidasius* strains. Calculation of the digital DNA-DNA hybridization (GGDC) [19] and OrthoANI [20] values for each paired combination of strains. The GGDC are the bottom and the OrthoANI the top values. (PDF 8 kb)

Additional file 3: Annotations of the protein families shared and unique among the compared *P. thermoglucosidasius* strains. The protein family datasets which are shared between different combinations of the four compared strains or unique to a particular strain were functionally annotated by RAST [36], comparison against the Conserved Domain Database [37] and classification according to their COG function using EggNOG mapper [36]. The proportions (%) of proteins (unique to strains or shared among different combinations of strains) belonging to each COG are graphically presented. (XLSX 484 kb)

Additional file 4: SNPs occurring in the CODH-NiFe group 4a locus genes of the compared *P. thermoglucosidasius* strains. The number of SNPs occurring in the individual CODH-NiFe group 4a genes of particular strains are indicated. The number in brackets indicates the number of non-synonymous amino acid substitutions observed in the amino acid sequence alignments for each individual gene. (XLSX 9 kb)

## Abbreviations

ANI: Average nucleotide identity
CO: Carbon monoxide
CO_2_: Carbon dioxide
CODH: Carbon monoxide dehydrogenase
COG: Conserved orthologous groups
GC: Gas chromatography
GGDC: Genome-to-genome distance calculator
H_2_: Hydrogen
HUS: Hemicellulose utilization system
mLB: Modified Luria-Bertani
O_2_: Oxygen
OD_600_: Optical density at 600 nm
SNP: Single nucleotide polymorphisms
TFBSs: Transcription factor binding sites
WGS: Water-gas shift

## References

[1] Aliyu H, Lebre P, Blom J, Cowan D, De Maayer P (2016). Phylogenomic re-assessment of the thermophilic genus Geobacillus. Syst Appl Microbiol.

[2] Zeigler DR (2014). The Geobacillus paradox: why is a thermophilic bacterial genus so prevalent on a mesophilic planet?. Microbiology.

[3] De Maayer P, Brumm PJ, Mead DA, Cowan DA (2014). Comparative analysis of the *Geobacillus* hemicellulose utilization locus reveals a highly variable target for improved hemicellulolysis. BMC Genomics.

[4] Shahinyan G, Margaryan A, Panosyan H, Trchounian A (2017). Identification and sequence analyses of novel lipase encoding novel thermophillic bacilli isolated from Armenian geothermal springs. BMC Microbiol.

[5] Thebti W, Riahi Y, Belhadj O. Purification and characterization of a new thermostable, Haloalkaline, solvent stable, and detergent compatible serine protease from Geobacillus toebii strain LBT 77. BioMed Res Int. 2016;2016:1–8. 10.1155/2016/9178962 PMC4812217 27069928

[6] Cripps RE, Eley K, Leak DJ, Taylor M, Todd M, Boakes S, Martin S, Atkinson T (2009). Metabolic engineering of *Geobacillus thermoglucosidasius* for high yield ethanol production. Metab Eng.

[7] Hussein AH, Lisokwska BK, Leak DJ (2015). The genus Geobacillus and their biotechnological potential. Adv Appl Microbiol.

[8] Valladares Juárez AG, Rost G, Heitmann U, Heger E, Müller R (2011). Development of a biotechnological process for the production of high quality linen fibers. Bioprocess Biosyst Eng.

[9] Omokoko B, Jäntges UK, Zimmermann M, Reiss M, Hartmeier W (2008). Isolation of the phe-operon from *G. stearothermophilus* comprising the phenol degradative meta-pathway genes and a novel transcriptional regulator. BMC Microbiolo.

[10] Tourova TP, Nazina TN, Mikhailova EM, Rodionova TA, Ekimov AN, Mashukova AV, Poltaraus AB (2008). alkB homologs in thermophilic Bacteria of the genus *Geobacillus*. Mol Biol.

[11] Brumm P, Land ML, Hauser LJ, Jeffries CD, Chang YJ, Mcad DA (2015). Complete genome sequence of *Geobacillus* strain Y4.1MC1, a novel CO-utilizing *Geobacillus thermoglucosidasius* strain isolated from Bath hot spring in Yellowstone National Park. Bioenergy Res.

[12] Mohr T, Aliyu H, Küchlin R, Polliack S, Zwick M, Neumann A, Cowan D, de Maayer P (2018). CO-dependent hydrogen production by the facultative anaerobe *Parageobacillus thermoglucosidasius*. Microb Cell Factories.

[13] Jacobson MZ (2009). Review of solutions to global warming, air pollution, and energy security. Energy Environ Sci.

[14] Evans A, Strezov V, Evans JT (2009). Assessment of sustainability indicators for renewable energy technologies. Renew Sust Energ Rev.

[15] Jain IP (2009). Hydrogen the fuel for 21st century. Int J Hydrog Energy.

[16] Rahman SNA, Masdar MS, Rosli MI, Majilan EH, Husaini T, Kamarudin SK, Daud WRW (2016). Overview biohydrogen technologies and application in fuel cell technology. Renew Sust Energ Rev.

[17] Nikolaidis P, Poullikkas A (2017). A comparative overview of hydrogen production processes. Renew Sust Energ Rev.

[18] Tirado-Acevedo O, Chinn MS, Grunden AM (2010). Production of biofuels from synthesis gas using microbial catalysts. Adv Appl Microbiol.

[19] Meier-Kolthoff JP, Auch AF, Klenk HP, Göker M (2013). Genome sequence-based species delimitation with confidence intervals and improved distance functions. BMC Bioinformatics.

[20] Lee I, Ouk Kim Y, Park SC, Chun J (2016). OrthoANI: an improved algorithm and software for calculating average nucleotide identity. Int J Syst Evol Microbiol.

[21] Suzuki Y, Kishigami T, Inoue K, Mizoguchi Y, Eto N, Takagi M, Abe S (1983). *Bacillus thermoglucosidasius* sp. Nov. a new species of obligately thermophilic bacilli. Int J Syst Evol Microbiol.

[22] Emms DM, Kelly S (2015). OrthoFinder: solving fundamental biases in whole genome comparisons dramatically improves orthogroup inference accuracy. Genome Biol.

[23] Eitinger T, Mandrand-Berthelot M-A (2000). Nickel transport systems in microorganisms. Arch of Microbiol.

[24] Bengtsson J, Tjalsma H, Rivolta C, Hederstedt L (1999). Subunit II of *Bacillus subtilis* cytochrome *c* oxidase is a lipoprotein. J Bacteriol.

[25] Andrews S, Mattatall NR, Arnold D, Hill BC (2005). Expression, purification, and characterization of the CuA–cytochrome c domain from subunit II of the Bacillus subtilis cytochrome caa3 complex in *Escherichia coli*. Protein Expr Purif.

[26] van der Oost J, von Wachenfeldt C, Hederstedt L, Saraste M (1991). *Bacillus subtilis* cytochrome oxidase mutants: biochemical analysis and genetic evidence for two *aa*_3_-type oxidases. Mol Microbiol.

[27] Solovyev V, Salamov A, Li RW (2011). Automatic annotation of microbial genomes and metagenomic sequences. Metagenomics and its applications in agriculture, biomedicine and environmental studies.

[28] Darling AE, Mau B, Perna NT (2010). progressiveMauve: multiple genome alignment with gene gain, loss and rearrangement. PLoS One.

[29] Tfsitescan: http://www.ifti.org/Tfsitescan. Accessed 20 Apr 2018.

[30] Inaoka T, Wang G, Ochi K (2009). ScoC regulates bacilysin production at the transcription level in *Bacillus subtilis*. Journal of Bacteriol.

[31] Kallio PT, Fagelson JE, Hoch JA, Strauch MA (1991). The transition state regulator Hpr of *Bacillus subtilis* is a DNA-binding protein. J Biol Chem.

[32] Kodgire P, Rao KK (2009). *hag* expression in *Bacillus subtilis* is both negatively and positively regulated by ScoC. Microbiology.

[33] Bankevich A, Nurk S, Antipov D, Gurevich AA, Dvorkin M, Kulikov AS, Lesin VM, Nikolenko SI, Pham S, Prjibelski AD, Pyshkin AV, Sirotkin AV, Vyahhi N, Tesler G, Alekseyev MA, Pevzner PA (2012). SPAdes: a new genome assembly algorithm and its applications to single-cell sequencing. J Comput Biol.

[34] Bosi E, Donati B, Galardini M, Brunetti S, Sagot MF, Lió P, Crescenzi P, Fani R, Fondi M (2015). MeDuSa: a multi-draft based scaffolder. Bioinformatics.

[35] Chen K, Lio C, Huang S, Shen H, Shieh Y, Chiu H, Lu C (2017). CSAR: a contig scaffolding tool using algebraic rearrangements. Bioinformatics.

[36] Overbeek R, Olson R, Pusch GD, Olsen GJ, Davis JJ, Disz T, Edwards RA, Gerdes S, Parrello B, Shukla M, Vonsetin V, Wattam AR, Xia F, Stevens R (2014). The SEED and the rapid annotation of microbial genomes using subsystems technology (RAST). Nucleic Acids Res.

[37] Zhou Y, Liang Y, Lynch KH, Dennis JJ, Wishart DS (2011). PHAST: a fast phage search tool. Nucleic Acids Res.

[38] Huerta-Cepas J, Szklarczyk D, Forslund K, Cook H, Heller D, Walter MC, Rattei T, Mende DR, Sunagawa S, Kuhn M, Jensen LJ, von Mering C, Bork P (2016). eggNOG 4.5: a hierarchical orthology framework with improved functional annotations for eukaryotic, prokaryotic and viral sequences. Nucleic Acids Res.

[39] Marchler-Bauer A, Bryant SH (2004). CD-search: protein domain annotations on the fly. Nucleic Acids Res.

[40] Thompson JD, Higgins DG, Gibson TJ (1994). Clustal W: improving the sensitivity of progressive multiple sequence alignment through sequence weighting, position-specific gap penalties and weight matrix choice. Nucleic Acids Res.

[41] Hall TA (1999). BioEdit: a user-friendly biological sequence alignment editor and analysis program for windows 95/98/NT. Nucleic Acids Symp Ser.

