# Supplementary figures and images for: Comparative genomic analysis of Parageobacillus thermoglucosidasius strains with distinct hydrogenogenic capacities
Source: BMC Genomics. 2018 Dec 6;19:880. doi: 10.1186/s12864-018-5302-9 (PMC6282330; doi:10.1186/s12864-018-5302-9)

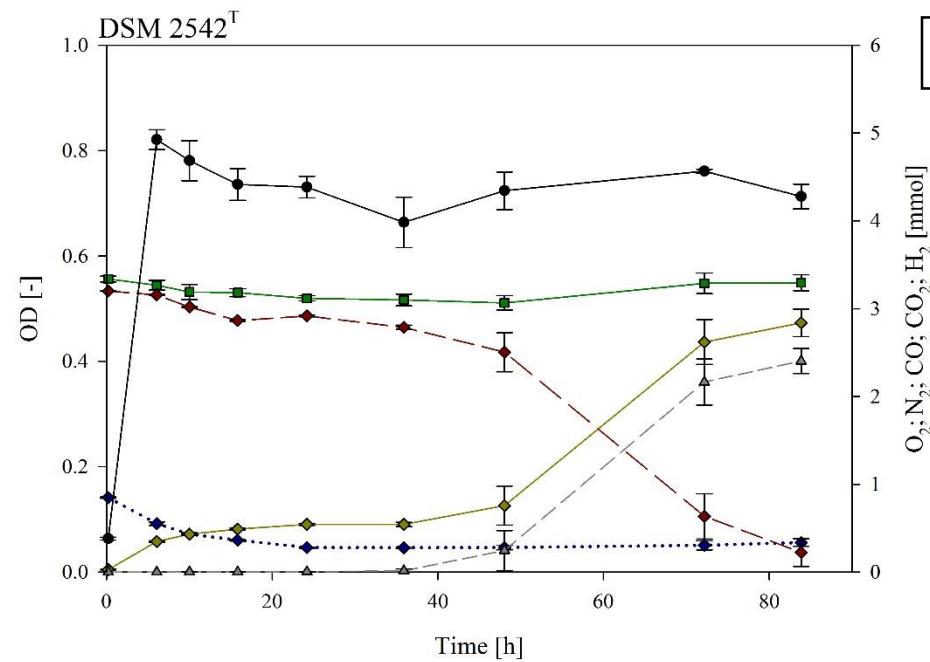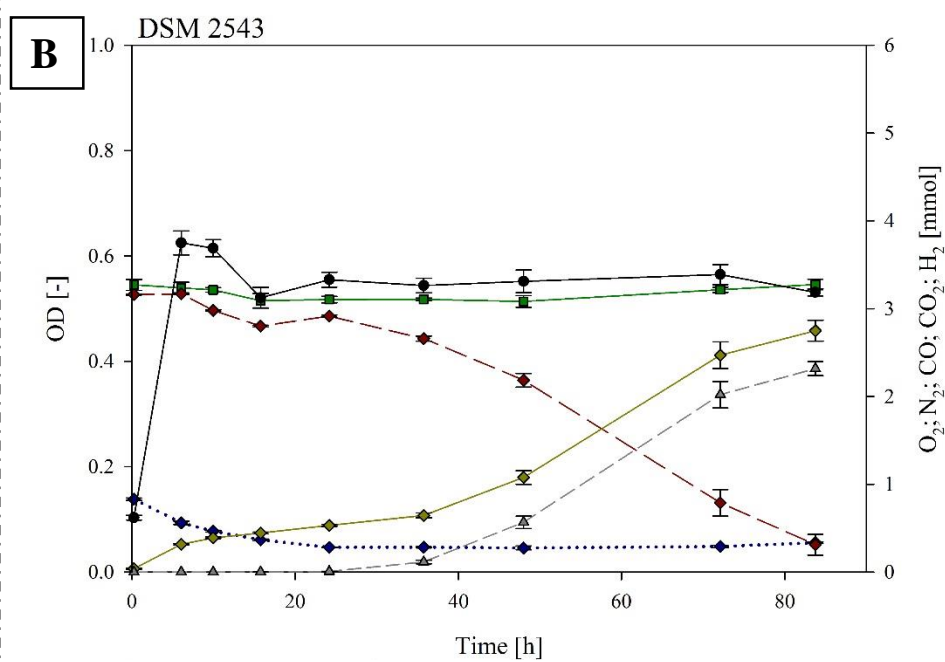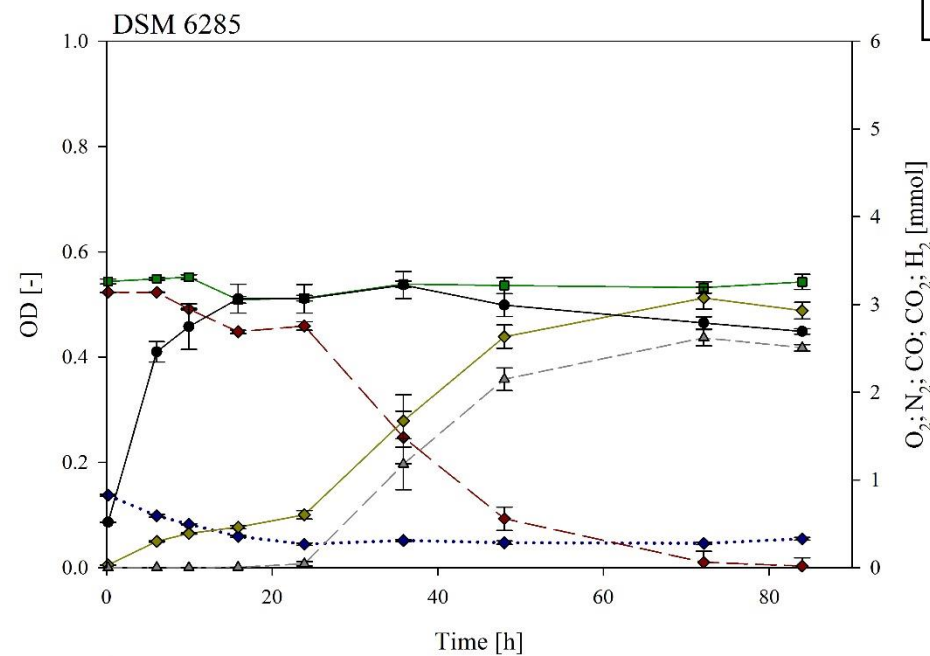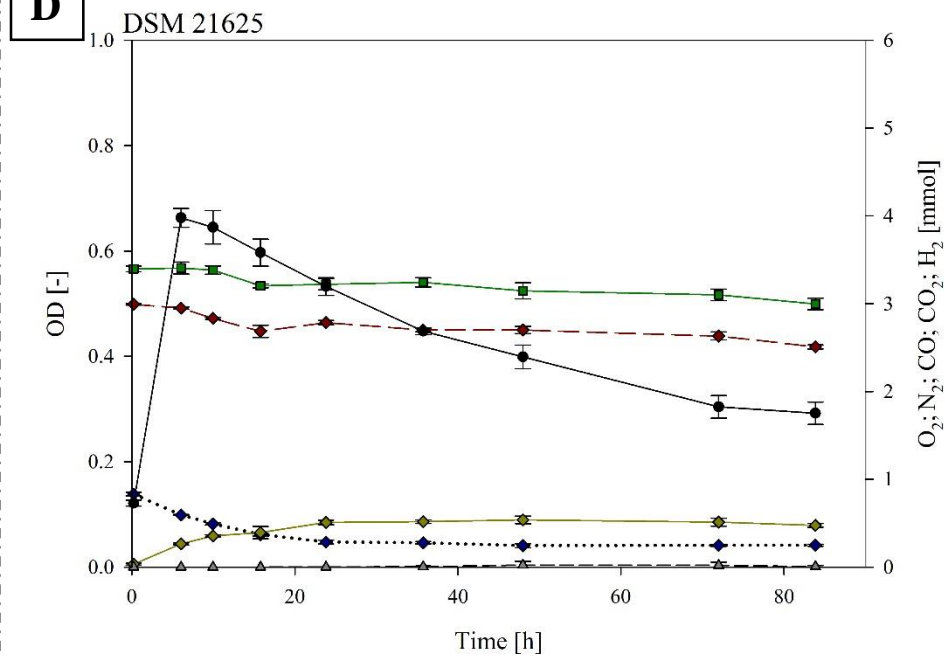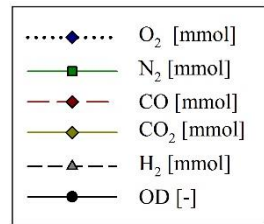

Supplement: Supplementary file 1 — Growth curve and gas composition during the cultivation of P. thermoglucosidasius), DSM 2542T (A), DSM 2543 (B) and DSM 6285 (C), DSM 21625 (D). All strains were cultivated in quadruplicate in mLB medium with an initial gas atmosphere consisting of 50% CO and 50% air for 84 h. (PDF 261 kb) [file 12864_2018_5302_MOESM1_ESM.pdf]
