# Supplementary material for: Comparative genomic analysis of Parageobacillus thermoglucosidasius strains with distinct hydrogenogenic capacities
Source: BMC Genomics. 2018 Dec 6;19:880. doi: 10.1186/s12864-018-5302-9 (PMC6282330; doi:10.1186/s12864-018-5302-9)
Supplement: Supplementary file 2 — Genomic relatedness among the four compared P. thermoglucosidasius strains. Calculation of the digital DNA-DNA hybridization (GGDC) [19] and OrthoANI [20] values for each paired combination of strains. The GGDC are the bottom and the OrthoANI the top values. (PDF 8 kb) [file 12864_2018_5302_MOESM2_ESM.pdf]

	**DSM 2542^{T}**	**DSM 2543**	**DSM 6285**	**DSM 21625**
**DSM 2542^{T}**	---	99.99%	99.24%	99.20%
**DSM 2543**	97.3%	---	99.27%	99.18%
**DSM 6285**	93.5%	93.6%	---	99.14%
**DSM 21625**	93.1%	93.1%	92.8%	---

OrthoANI

GGDC
